# Molecules and Biomaterial Technologies Affecting Stem Cell Differentiation

**DOI:** 10.1155/2022/9783430

**Published:** 2022-04-16

**Authors:** Lorenzo Lo Muzio, Marco Mascitti, Marcella La Noce, Francesca Posa, Yasusei Kudo, Nicola Cirillo

**Affiliations:** ^1^University of Foggia, Foggia, Italy; ^2^Università Politecnica Delle Marche, Ancona, Italy; ^3^Università Della Campania, Napoli, Italy; ^4^Tokushima University, Tokushima, Japan; ^5^University of Melbourne, Melbourne, Australia

Mesenchymal Stem Cells (MSCs) are multipotent cells able to differentiate into specialized cells developing from mesoderm and to regenerate different tissues [1].

These adult stem cells were originally identified in the bone marrow, which is still considered the best cell source, but can also be isolated from several adult tissues such as adipose tissue, dental tissues, skin, brain, liver, and fetal tissues [2].

MSCs manifest peculiar stem cell properties of self-renewal and multipotency.

It has been extensively demonstrated that MSCs can be induced to differentiate *in vitro* into different cell types: not only mesodermal lineage cells, such as osteocytes, chondrocytes, and adipocytes, but also endothelial cells or hepatocytes [3]. Furthermore, MSC survival and differentiation towards a specific cell line can be influenced by molecular or physical factors.

Studies have convincingly demonstrated that MSCs are capable of repairing damaged tissues, particularly when appropriate microenvironmental conditions are present. This unique ability confers MSCs a tremendous potential for innovative therapeutic approaches, such as regenerative medicine, for the treatment of illness or disabilities [4].

This special issue highlights the most recent research progresses on factors, molecules, or stimuli derived from the extracellular microenvironment, which could affect MSCs' fate and commitment. Moreover, it includes a historical review about stem cell usage that also highlights their biological, religious, and ethical implications.

Bone regenerative medicine can exploit different strategies; among them, the most common approach consists in the use of MSCs grown on biocompatible scaffolds able to mimic their natural environment [5]. Often, these scaffolds are combined with factors that facilitate proliferation and osteogenic differentiation processes of MSCs [6, 7]. Nonetheless, there are several limitations associated with this type of methods [8]. A valid alternative is constituted by scaffolds coated with compounds similar to extracellular matrix components [9] and chemokines capable of inducing the recruitment, the adhesion, and the differentiation of MSCs to the damaged site [10]. In this line, stromal cell-derived factor-1*α* (SDF-1*α*) has been described as one of the key chemokines in promoting site-specific migration of healing MSCs [11].

Interestingly, L. Li et al. have demonstrated the ability of the microporous composites, developed combining OPF/BP with SDF-1*α*, in promoting migration and osteogenic differentiation of rat bone marrow MSCs (BMSCs), which makes it a good material useful to increase bone regeneration.

MSC fate can be influenced not only by molecular factors but also by mechanical stimuli [12]. Several cellular mechanical loading models have been developed with the purpose of studying cell mechanoresponses.

In this regard, Y. Zhao et al. have authored a detailed article dedicated to the development of a novel device, called iStrain. It is an elastic membrane specifically designed to apply mechanical tension on human BMSCs in culture. The authors have demonstrated the capacity of this device in promoting BMSC differentiation toward the osteogenic and fibrogenic lineages.

The strategy based on the use of MSCs cultured on scaffolds is also extensively studied for its possible applications in cartilage tissue engineering [13]. Porous honeycomb-like sheets made of polylactic acid (PLA) are the most widely used scaffolds in this regard, although the optimal pore size has not yet been identified to ensure efficient cell adhesion and cartilage formation. M. Yagi et al., in their research, have proved the capacity of PLA honeycomb films in prompting cartilage formation starting from human synovial MSCs and identifying the 5 *μ*m pores as the best dimension for *in vitro* cartilage formation.

Adipose tissue-derived MSCs (AT-MSCs) represent another attractive model of MSCs for cartilage tissue engineering. An innovative procedure for generating cartilage-like tissue is proposed by Q. T. Dang et al. The method presented is based on the use of AT-MSCs grown on a human dermal collagen matrix obtained from human skin, which could serve as a very reliable bioscaffold. This technical solution could also be used with other stem cells of mesenchymal origin.

These reports are really important in shaping our understanding of the mechanisms driving MSC differentiation *in vitro* with the purpose of identifying innovative therapeutic approaches for the regeneration of connective tissues as bone and cartilage.

In summary, these papers put together the most recent findings about molecules and materials suitable for MSC commitment and differentiation, very useful for clinicians and basic scientists.

*Lorenzo Lo Muzio* *Marco Mascitti* *Marcella La Noce* *Francesca Posa* *Yasusei Kudo* *Nicola Cirillo*
